# Tooth replacement in the early-diverging neornithischian *Jeholosaurus shangyuanensis* and implications for dental evolution and herbivorous adaptation in Ornithischia

**DOI:** 10.1186/s12862-024-02233-2

**Published:** 2024-04-16

**Authors:** Jinfeng Hu, Xing Xu, Fuqiang Li, Fenglu Han

**Affiliations:** 1 School of Earth Sciences, China University of Geosciences, 388 Lumo Road, 430074 Wuhan, Hubei Province China; 2 Center for Vertebrate Evolutionary Biology, Yunnan University, Kunming, China; 3 Key Laboratory of Vertebrate Evolution and Human Origins, Institute of Vertebrate Paleontology and Paleoanthropology, Chinese Academy of Sciences, Beijing, China; 4 Yifu Museum of China University of Geosciences, Wuhan, Hubei China

**Keywords:** Ornithischia, Ornithopoda, Jehol Biota, Tooth replacement, Cretaceous

## Abstract

**Background:**

Tooth replacement patterns of early-diverging ornithischians, which are important for understanding the evolution of the highly specialized dental systems in hadrosaurid and ceratopsid dinosaurs, are poorly known. The early-diverging neornithischian *Jeholosaurus*, a small, bipedal herbivorous dinosaur from the Early Cretaceous Jehol Biota, is an important taxon for understanding ornithischian dental evolution, but its dental morphology was only briefly described previously and its tooth replacement is poorly known.

**Results:**

CT scanning of six specimens representing different ontogenetic stages of *Jeholosaurus* reveals significant new information regarding the dental system of *Jeholosaurus*, including one or two replacement teeth in nearly all alveoli, relatively complete tooth resorption, and an increase in the numbers of alveoli and replacement teeth during ontogeny. Reconstructions of Zahnreihen indicate that the replacement pattern of the maxillary dentition is similar to that of the dentary dentition but with a cyclical difference. The maxillary tooth replacement rate in *Jeholosaurus* is probably 46 days, which is faster than that of most other early-diverging ornithischians. During the ontogeny of *Jeholosaurus*, the premaxillary tooth replacement rate slows from 25 days to 33 days with similar daily dentine formation.

**Conclusions:**

The tooth replacement rate exhibits a decreasing trend with ontogeny, as in *Alligator*. In a phylogenetic context, fast tooth replacement and multi-generation replacement teeth have evolved at least twice independently in Ornithopoda, and our analyses suggest that the early-diverging members of the major ornithischian clades exhibit different tooth replacement patterns as an adaption to herbivory.

**Supplementary Information:**

The online version contains supplementary material available at 10.1186/s12862-024-02233-2.

## Background

During the Mesozoic, ornithischians evolved high-fiber herbivory several times and exhibited high levels of morphological disparity [1], allowing analysis of common phenotypic responses to herbivory [2]. However, previous studies regarding ornithischian dietary specialization mostly concentrate on tooth morphology [3], biomechanical modeling [2, 4–6], and dental histology [7, 8]. In hadrosaurid and ceratopsid dinosaurs, dental batteries are composed of a large number of teeth that are interlocked vertically and rostrocaudally and are also inferred to be specializations for processing tough plant material [9], which is considered key to their radiation [7]. The formation of these dental batteries includes changes to tooth replacement patterns, such as an increase in the number of replacement teeth and the locations of the replacement teeth [10]. However, after Edmund [11] surveyed the tooth replacement pattern of vertebrates, the evolution of tooth replacement patterns and its relationship to herbivorous adaptations in ornithischians except for some work on hadrosaurids and ceratopsids [7, 8] have not yet been studied in detail.

*Jeholosaurus shangyuanensis* is a small neornithischian from the Lower Cretaceous Yixian Formation in Liaoning Province, China [12]. Previous research focused on skeletal morphology [13, 14] and bone histology [15]. The phylogenetic position of *Jeholosaurus* remains debated and the main dispute is whether *Jeholosaurus* is a non-cerapodan ornithischian [16, 17] or an early-diverging ornithopod [18, 19]. Brown, Butler [20] suggested that the matrix from Boyd [17] was far more susceptible to the influences of both character and taxon sampling and the matrices from Han, Forster [18], Dieudonné, Cruzado-Caballero [19] are more stable. Hence, we place *Jeholosaurus* as an early-diverging ornithopod following Han, Forster [18]. Here, we investigate the tooth replacement pattern and replacement rate in *Jeholosaurus* using micro-CT imaging and dental histology. We also collected data on the replacement patterns in other ornithischians and analyzed them in a phylogenetic context, providing new information on replacement patterns in early-diverging neornithischians and helping to improve our understanding of the evolution of these patterns with respect to high-fiber herbivory.

## Materials and methods

### Material

**Fig. 1:**
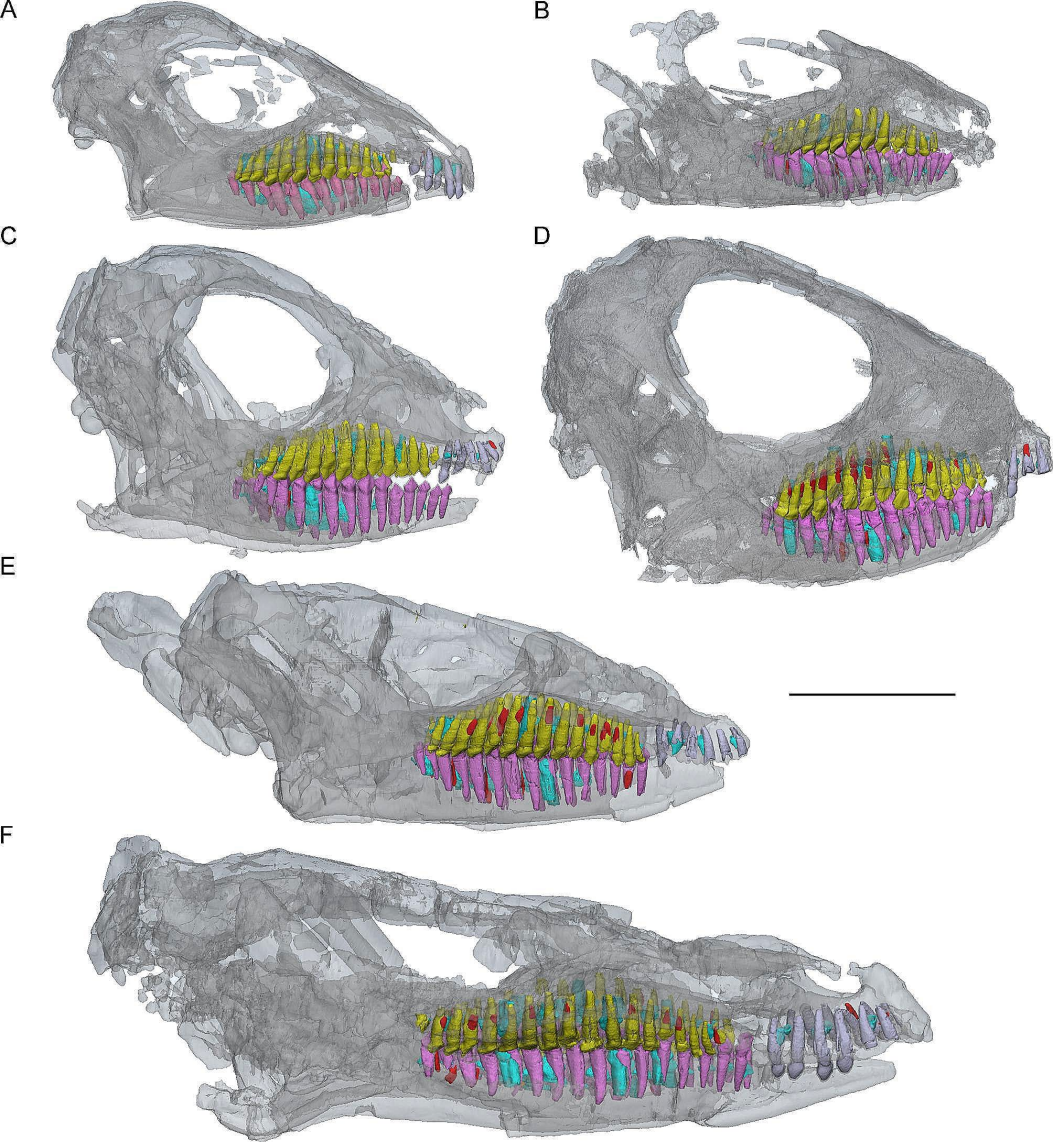
3D models of skulls in right transparent view of six *Jeholosaurus* specimens. (**A**) CUGW VH132 (early juvenile); (**B**) IVPP V15719 (early juvenile); (**C**) IVPP V12530 (late juvenile); (**D**) IVPP V15718 (late juvenile); (**E**) IVPP V12529 (late juvenile); (**F**) IVPP V15717 (subadult). CT reconstructions are color coded as follows: functional premaxillary teeth, violet; functional maxillary teeth, yellow; functional dentary teeth, lavender; replacement teeth, cyan; remnants of resorbed functional teeth, red. Scale bar equals 20 mm

Seven individuals of *J. shangyuanensis* are included in this study: IVPP V12529 (the holotype), IVPP V12530, IVPP V15717, IVPP V15718, IVPP V15718, CUGW VH132, and YLSNHM01797 (Fig. 1). The fossil material used in this study, except CUGW VH132 and YLSNHM01797, is housed in the Institute of Vertebrate Paleontology and Paleoanthropology, Chinese Academy of Sciences, Beijing, China (IVPP) and CUGW VH132 (Collection number: Ei32RH) is housed in the Yifu Museum of China University of Geosciences, Wuhan, Hubei, China. YLSNHM01797 is accessioned at the public institute Yinliang Stone Natural History Museum, Fujian Province, China.

**CUGW VH132**. This new specimen consists of a complete skull, the complete left and right femora, the complete left tibia and the proximal part of the right tibia, the complete left fibula and the proximal part of the right fibula, the left metatarsals, the left and right ilium, the left and right ischium, six sacral vertebrae, and seven caudal vertebrae. The skull is the smallest known for the species, with a length of about 49.50 mm. We assigned this specimen to *Jeholosaurus* based on the following character states: the presence of a row of small foramina on the lateral surface of the nasal immediately dorsal to the premaxillary articulation; the presence of a foramen enclosed within the quadratojugal; and jugal caudal process bifurcated distally [13, 14]. However, the premaxillae only contain five teeth, differing from other individuals. This may be interpreted as an ontogenetic change. The dentitions of the premaxillae, maxillae, and dentary have been reconstructed.

**IVPP V15719**. The incomplete skull length is about 45.33 mm due to the absence of the premaxillae. The right femur length (49 mm) and tibia length (65 mm) are slightly larger than CUGW VH132. The snout is damaged, and the braincase and the skull table are largely absent. The complete maxillary and dentary dentitions have been reconstructed.

**IVPP V12530**. The cranial elements of this specimen appear to be complete and articulated although the premaxillae and the left dentary are crushed. The entire skull is slightly compressed transversely. Skull length is about 55.20 mm. The dentitions of the premaxillae, maxillae, and dentary have been reconstructed.

**IVPP V15718**. The skull and mandible are preserved completely and slightly compressed left laterally. Skull length is about 58.63 mm. Only three premaxillary teeth are preserved. The dentitions of the maxillae and dentary have been reconstructed.

**IVPP V12529**. This specimen has an almost complete skull and mandible. The skull is compressed dorsoventrally with the skull roof displaced. Skull length is about 71.50 mm (measured from the tip of the snout to the caudal margin of the quadrate). The dentitions of the premaxillae, maxillae, and dentary are reconstructed.

**IVPP V15717**. The individual has the largest skull known with a length of 97.90 mm. It preserves nearly complete cranial elements but has been seriously compressed dorsoventrally. The frontal and nasal are displaced. The dentitions of the premaxillae, maxillae, and dentary have been reconstructed.

**YLSNHM01797.** The individual has a complete skull, partial mandible, and most postcranial skeleton. The skull length is about 79.70 mm.

Histological analysis suggests that there are no lines of arrested growth (LAGs) existing in the tibia and fibula of IVPP V15719 and it is an early juvenile [15]. The holotype (IVPP V12529) preserves at least two LAGs and is regarded as a late juvenile [15]. IVPP V15717 has a larger skull than IVPP V20379 which is divided into subadult stage by Han, Zhao [15] and is also regarded as a subadult. The ontogenetic stages of specimens described here are listed in Table 1.

### Computed tomography

The roots and the replacement teeth are normally concealed in the jaws. By employing traditional methods, it is difficult to obtain the internal anatomical features of the dentitions in any detail. The advent of noninvasive and nondestructive radiological approaches, such as X-ray computed tomography (CT), has revolutionized the study of fossil specimens [21], providing new insights into internal structures normally obscured by bones and rock matrix.

Here, we conducted high-resolution X-ray micro-CT on these specimens and reconstructed 3D models of the dentitions. Scanning of IVPP V12529, IVPP V12530, IVPP V15717, IVPP V15718, and IVPP V15719 was carried out using a customized 225 kV micro-CT instrument (225 Micro CT) at the Key Laboratory of Vertebrate Evolution and Human Origins of the Chinese Academy of Sciences, Beijing, China. Scanning on CUGW VH132 was carried out using a 300 kV micro-computed tomography instrument (Phoenix Vtomex M) and the detector (Dynamic 41–100) at the Shanghai Yinghua Inspection and Testing Co., Ltd, Shanghai, China. The scanning parameters of these specimens are displayed in Table 1.

**Table 1:** Skull length and scanning parameters of *Jeholosaurus*

Specimen number	Skull length* (mm)	Scanning voltage (kV)	Scanning current (µA)	Resolution (µm)	Growth stage
CUGW VH132	49.50	240	250	60.30	Early juvenile
IVPP V15719	45.33↑	140	100	34.50	Early juvenile
IVPP V12530	55.20	140	100	57.24	Late juvenile
IVPP V15718	58.63	140	100	43.91	Late juvenile
IVPP V12529	71.50	140	100	54.81	Late juvenile
IVPP V15717	97.90	140	100	61.95	Subdult

*Skull length is measured from the tip of the snout to the caudal margin of the quadrate

### The reconstruction of Zahnreihen

Tooth replacement is a continuous progress occurring throughout the life of reptiles. Edmund [11] first studied the replacement patterns systematically. Teeth in most reptiles are replaced in an ordered, alternating segmented, and wave-like pattern along the jaw called a Zahnreihe [22]. Each Zahnreihe consists of a series of teeth including unerupted teeth, where the most rostrally positioned tooth is more mature than those situated caudally [23].

Some previous research inferred the replacement pattern by the degree of wear plus root resorption and the developmental stage of the pulp cavities [10, 24]. Here, we use the replacement index proposed by Fastnacht [25] to quantify the replacement stages independently of varying total tooth length. A fully-grown tooth without a replacement tooth is assigned a replacement index of 1.0. If a replacement tooth is present, its replacement index is calculated as the value of its total tooth length divided by the total tooth length of the corresponding functional tooth. The corresponding functional tooth is then given a replacement index of 1.0 plus the replacement index of its replacement tooth. The replacement index for each functional and replacement tooth can then be plotted on a graph whose *y*-axis is the replacement index and whose *x*-axis is the tooth position. These plots show that these teeth exhibit a regular pattern in which the replacement index decreases progressively and periodically over several tooth positions. Each degressive sequence represents a Zahnreihe, which is formed of a series of teeth that are linked with each other on the plot by solid lines. The distance between two successive Zahnreihen is referred to as the Z-spacing, which is measured as the distance from a tooth in the replacement wave to the adjacent Zahnreihen. The Z-spacing of *Jeholosaurus* is described from the means of all measurements.

### Ancestral state reconstruction (ASR)

We used ancestral state reconstructions to estimate the ancestral state of the replacement pattern in Ornithischia and examine changes to the replacement pattern in a phylogenetic context. All analyses were performed in R v. 4.1.3 [26]. We assembled manually a topology for all taxa used here in Mesquite v. 3.40 [27] based on previous research [18, 20, 28–30] with *Silesaurus* as the outgroup. Branch lengths were estimated based on geological ages, which were taken from the Paleobiology Database (https://www.paleobiodb.org). The time-calibrated tree was produced using the R package Strap [31] based on the ‘equal’ method and the function geoscalePhylo was used to plot the time-scaled tree against a geological time scale.

ASR for the tooth replacement pattern (a discrete trait) was performed in phytools [32] using Bayesian stochastic character mapping (SIMMAP). The list of tooth replacement patterns used for ASR can be seen in Additional File 1. SIMMAP is based on Bayesian posterior sampling of stochastic character maps using Monte–Carlo Markov Chain (MCMC), which can account for branch length information, rate heterogeneity, and phylogenetic uncertainty [33]. For ASR, we estimated Akaike weights [34] for each of the three candidate models for transition rates (equal, symmetrical, and all different rates) and generated stochastic maps in a proportion of the weight of each model out of 1000 simulations, following the method of Legendre, Choi [35].

### Thin sectioning

To calculate daily rates of dentine formation in *Jeholosaurus*, we thin-sectioned two teeth from YLSNHM01797: one premaxillary tooth crown and one maxillary tooth. In addition, an isolated premaxillary tooth was found in the matrix surrounding CUGW VH132. Based on its total tooth length and morphology, we suggest that it is the rPM5 of CUGW VH132. This isolated tooth was also thin-sectioned. The thin-sections of teeth were prepared following the procedure described by Erickson [36]. Each tooth was embedded in resin (Araldite 2020 epoxy resin) and then cut with an STX-202 A diamond wire cutting machine. The cut surface was sanded with grit abrasives (standard grades 400, 800, 1000) and polished to make thin-sections approximately 50–100 μm thick.

### Imaging and measurements

Images of the thin-sections were observed and traced using a Zeiss Primotech microscope at 10x or 20x magnification. In thin–sections, incremental lines of von Ebner are ubiquitous light/dark couplets that record the daily apposition of dentine in teeth [36]. The number of incremental lines of von Ebner indicates tooth formation time, whereas their thickness indicates the rate of dentine formation per day [37, 38]. Measurements from thin-sections were taken using ImageJ v. 1.53 [39] to get the daily dentine apposition rate (DDAR) and the counts of incremental lines. We followed the methods and arguments laid out in D’Emic, O’Connor [40] in identifying von Ebner’s incremental width.

## Results

### Premaxillary dentitions

**Fig. 2:**
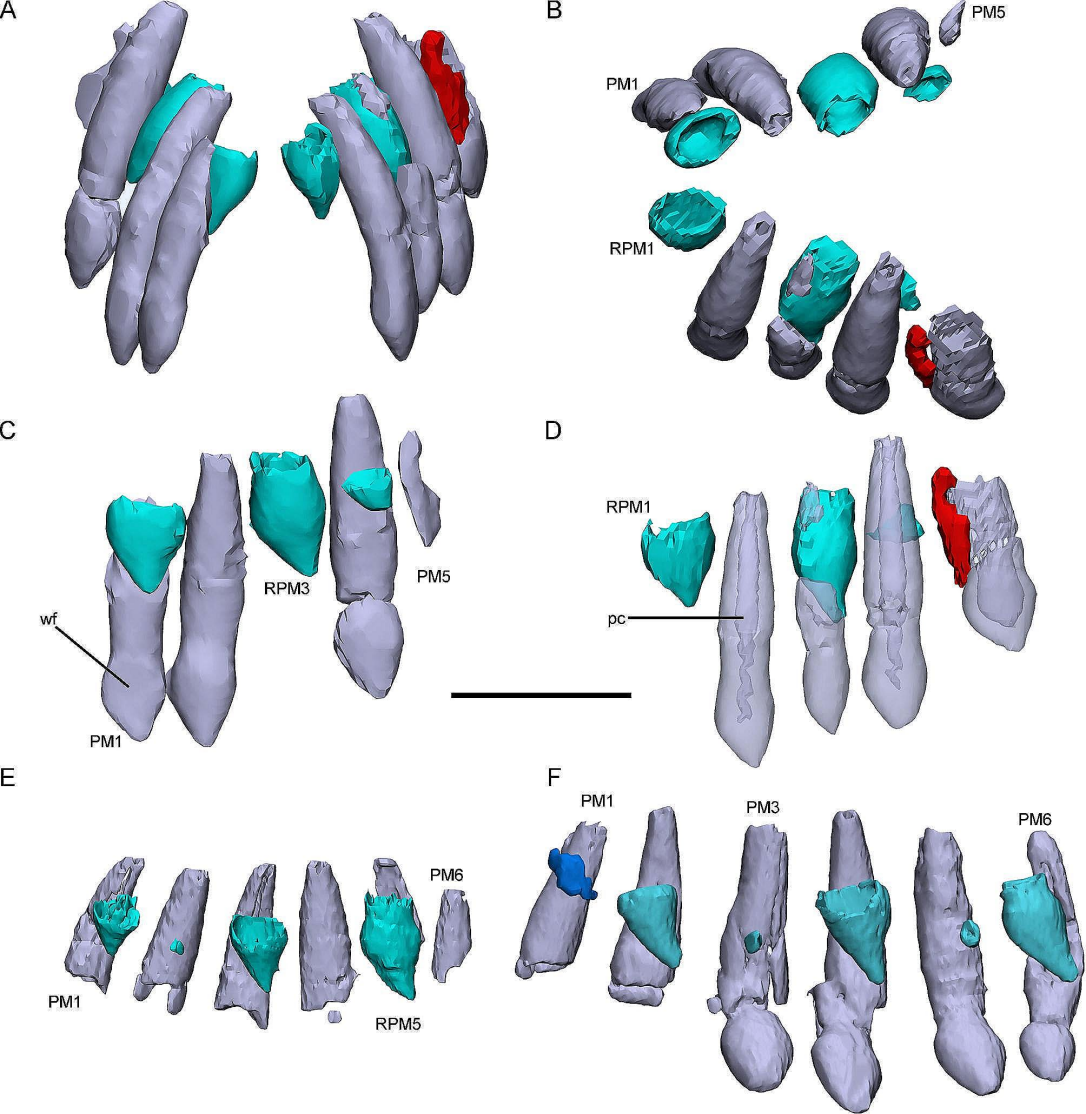
3D reconstructions of premaxillary dentitions in *Jeholosaurus*. Premaxillary dentitions in the early juvenile CUGW VH132 in rostral (**A**) and dorsal (**B**) view. Right premaxillary tooth row in CUGW VH132 in lingual view (**C**). Left premaxillary tooth row in CUGW VH132 in labial view (**D**). Right premaxillary tooth rows in the late juvenile IVPP V12529 (**E**) and the subadult IVPP V15717 (**F**) in lingual view. Elements in the CT reconstructions are color coded as follows: functional premaxillary teeth, violet; replacement teeth, cyan; remnants of resorbed functional teeth, red; a thin layer of dentine (OF? ), blue. Scale bar equals 5 mm (**A**-**D**) and 3 mm (**E**-**F**)

IVPP V15717, IVPP V12529, and CUGW VH132 have relatively complete premaxillae (Fig. 2). The premaxillae of the subadult specimen IVPP V15717 and the late juvenile IVPP V12529 contain six alveoli, whereas the smallest specimen CUGW VH132 only contains five on the left and right side (Fig. 2, S1). This suggests that one new premaxillary alveolus appeared during ontogeny.

In rostral view, the functional teeth are arched and incline lingually (Fig. 2A). The total length of functional teeth increases caudally but decreases in the last alveolus (Fig. 2D). In dorsal view, the tooth rows extend caudolaterally and the angle between them is about 30° (Fig. 2B). The digital reconstructions show that functional teeth bear nearly conical roots and bulbous crowns with tips that are curved distally (Fig. 2C). The functional roots expand slightly in their middle parts. In lingual view, the crown of rPM1 bears a slightly dull tip and a concave surface different from the other premaxillary crowns which are convex. Therefore, we suggest that the concave surface on the premaxillary crowns are wear facets (**wf**). In transparent view, the pulp cavity (**pc**) is narrow and open at its top (Fig. 2D).

Replacement teeth are well preserved in the early juvenile CUGW VH132, the late juvenile IVPP V12529, and the subadult IVPP V15717. The replacement teeth occur alternately in the premaxillary tooth rows (Fig. 2D-F, S1). In the early juvenile specimen CUGW VH132, three replacement teeth are preserved in each premaxilla (the 1st, 3rd, and 5th alveoli) (Fig. 2, S1). In the late juvenile IVPP V12529, four replacement teeth are present in the premaxilla (the 3rd to 5th alveoli on the left; 1st, 2nd, 3rd, and 5th on the right) (Fig. 2E). The subadult specimen IVPP V15717 has three replacement teeth on the left side (2nd, 4th, and 6th alveoli) and five replacement teeth on the right (2nd to 6th alveoli) (Fig. 2, S1). The replacement teeth are positioned lingual to the corresponding functional teeth (Fig. 2B). As in the functional teeth, the replacement crown tips also appear to curve distally (Fig. 2C). The replacement crowns have a rhomboidal outline in lingual view and some relatively mature replacement crowns are wider than their corresponding functional teeth crowns (Fig. 2D). Few replacement roots are preserved in the premaxillae but where present they are oval in cross-section (Fig. 2C and E, and 2F).

After being resorbed the functional teeth are shed, leaving a thin layer of dentine called the old resorbed tooth (Fig. 2D). All specimens except IVPP V12529 preserve the remnants of resorbed functional teeth. These remnants are normally positioned labiocaudal to the functional teeth and are irregularly shaped. In general, the remnants of resorbed teeth show no obvious pattern but they still provide reliable evidence for identifying replacement stages.

### Maxillary dentitions

Maxillary dentitions are well preserved in six specimens and the number of alveoli increases from 13 to 18 during ontogeny (Fig. S2). In the late juvenile IVPP V12530, the right maxilla preserves 13 functional teeth whereas the left maxilla has one more functional tooth. This extra tooth only preserves a partial crown and is positioned caudal to lM13 demonstrating that the tooth is newly germinated. This situation is similar to the replacement tooth growth and may explain how new teeth erupt.

**Fig. 3:**
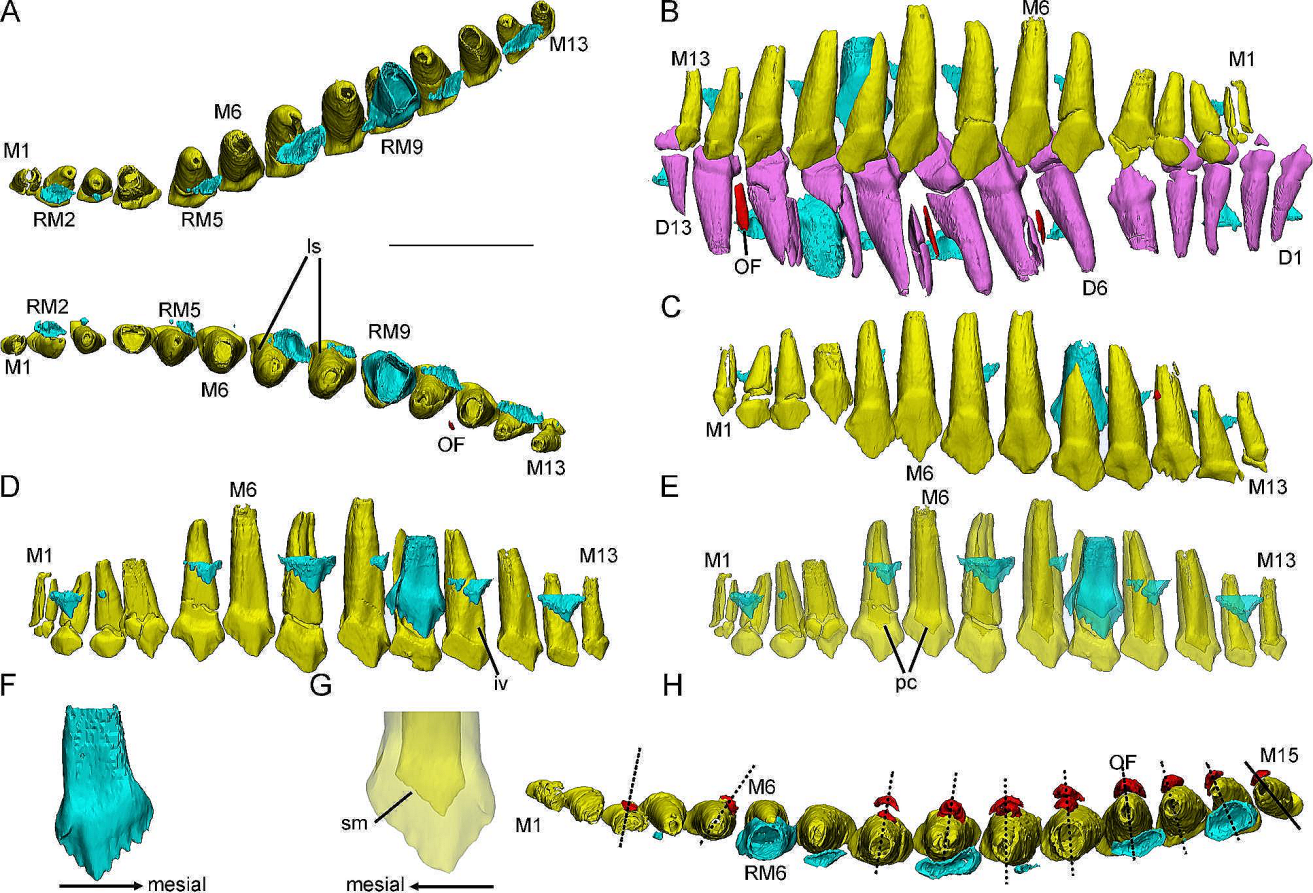
3D reconstructions of maxillary and dentary dentitions in the early juvenile IVPP V15719 (**A**-**G**) and the late juvenile IVPP V15718 (**H**). Maxillary dentitions in dorsal view (**A**). Right maxillary and dentary tooth rows in labial view (**B**). Left maxillary tooth row in labial view (**C**). Right maxillary tooth row in lingual (**D**) and transparent (**E**) view. lRM9 in lingual view (**F**). The crown of rM4 in lingual view (**G**). Right maxillary tooth row in dorsal view (**H**). Elements in the CT reconstructions are color coded as follows: functional maxillary teeth, yellow; replacement teeth, cyan; remnants of resorbed functional teeth, red. Scale bars equal 5 mm (**A**-**E**, **H**), 2 mm (**G**), and 3 mm (**F**)

In dorsal view, the maxillary tooth rows diverge caudolaterally forming an angle of about 30° with the sagittal plane (Fig. 3A). Generally, the total tooth length of the maxillary teeth increases to a maximum at M8 or M9, then decreases caudally, with the caudal-most tooth similar in size to M1 (Fig. 3B). Compared with the premaxillary teeth, most of the maxillary teeth are shorter and the functional teeth in the maxillae show no obvious trends in inclination or curvature (Fig. 3C-D). In lateral view, the maxillary tooth rows usually cover the dentary tooth rows and are closely packed with them (Fig. 3B).

Digital reconstructions show that the functional roots are nearly conical with subcircular cross-sections (Fig. 3C-D). As in some late-diverging ornithopods [41, 42] and ceratopsians [43–45], the roots in *Jeholosaurus* develop shallow longitudinal sulci (**ls**) on their mesial and distal surfaces which is thought to allow for closer packing of the dentitions in ceratopsians (Fig. 3A). Similar to the premaxillae, the pulp cavities (**pc**) are narrow at the roots and some are open apically (Fig. 3E). The pulp cavities at the base of the crowns are spade-like with some serrated margins similar to denticles (Fig. 3E and G). In general, the outline of the pulp cavity is similar to that of the functional tooth crown. In lingual view, an inverted V-shaped (**iv**) pit develops dorsal to the base of the crown (Fig. 3D).

The functional crowns are fan-like with the base swelling labially (Fig. 3C). In labial view, the functional crowns are asymmetric around the central axis and there are denticles on the mesial and distal margins (Fig. 3C). Some newly erupted functional teeth bear four to five denticles mesial to the primary ridges while the denticles distal to the primary ridge are only one to two. Compared with the dentary dentitions, the primary ridges are located more distally and are less prominent (Fig. 3B). In lingual view, an abrupt cingulum is present at the base of the crowns (Fig. 3D). Apical to the cingulum a trapezoidal wear facet develops mesial to the primary ridges. In some functional teeth at a late replacement stage, the wear facets are well developed and the primary ridges have been worn away (Fig. 3C-D).

Maxillary replacement teeth are well preserved in all specimens and the general trend of the number of the replacement teeth per jaw is increasing with the ontogeny of *Jeholosaurus* (Fig. S2). A second generation of replacement teeth is only observed in the subadult specimen (IVPP V15717) (the 10th alveolus in the left maxilla). The replacement teeth newly formed only develop small tips (Fig. 3D). The replacement crowns are similar to their corresponding functional teeth but have more prominent denticles. The medial and distal carinae are asymmetrical with the former slightly longer. Approximately five and three denticles are distributed over the mesial and distal carinae, respectively, and all denticles are subequal in size (Fig. 3F). As the replacement teeth develop, they gradually move basally but are still located basal to the cingulum. In dorsal view, the replacement teeth bear thinner dentine and larger pulp cavities than the corresponding functional tooth (Fig. 3A).

In the early juvenile IVPP V15719, only one remnant of a resorbed tooth is preserved labial to the functional tooth in the 11th alveolus (Fig. 3A). lM11 might be newly erupted because it bears clear mesial denticles and no resorption pits (Fig. 3C and E), so preserving a small remnant of the previous tooth. In other specimens, many remnants are preserved. Among them, the late juveniles IVPP V15718 and IVPP V12529 bear a second generation of remnants located ventrolabial to the first generation remnants (Fig. 3H). Similar to the premaxillary dentitions, the remnants of resorbed teeth in the maxilla are also composed of a thin layer of dentine. In dorsal view, the remnants in the rostral part of the tooth rows are positioned more distally whereas remnants in the caudal part are positioned more mesially, as also occurs in *Liaoceratops* [45]. The tracts of partially resorbed teeth are thought to track the growth of the jaws (Fig. 3H) [45].

### Dentary dentitions

**Fig. 4:**
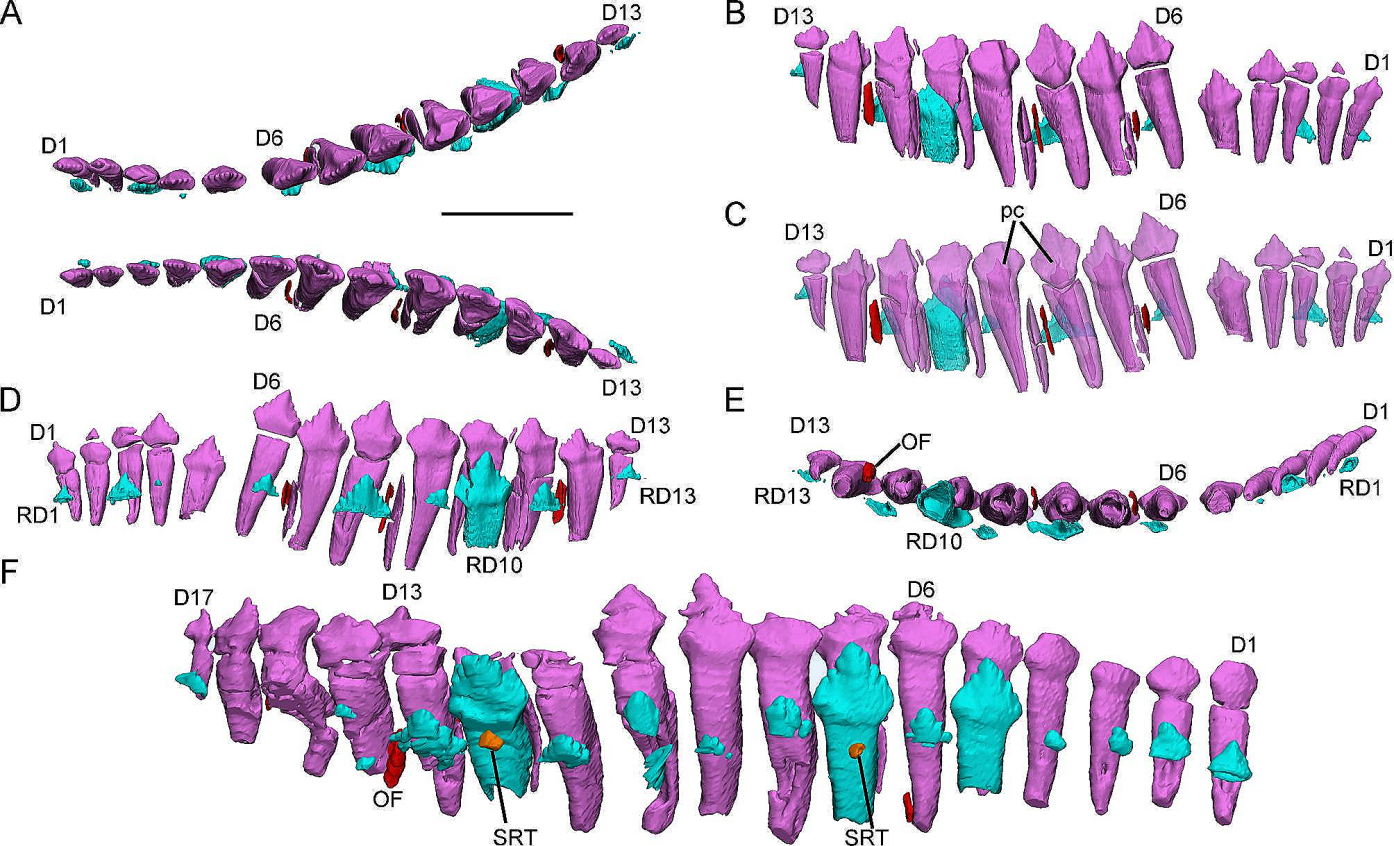
3D reconstructions of dentary dentitions in the early juvenile IVPP V15719 (**A**-**E**) and the subadult IVPP V15717 (**F**). Dentary dentitions in dorsal view (**A**). Right dentary tooth row in labial (**B**), labial transparent (**C**), lingual (**D**), dorsal (**E**) view. Left dentary tooth row in lingual view (**F**). Elements in the CT reconstructions are color coded as follows: functional dentary teeth, lavender; replacement teeth, cyan; remnants of resorbed functional teeth, red; the second generation of replacement teeth, orange. Scale bar equals 5 mm

All specimens preserve relatively complete dentaries except for IVPP V12530 whose left dentary is broken rostrally. The number of alveoli increases from 13 to 17 during ontogeny (rather than 18 s in the maxillary dentitions) (Fig. S3). In the dentary of *Jeholosaurus*, functional tooth size increases to a maximum at D8 or D9 similar to the maxillary dentitions (Fig. 4).

In labial view, the long axes of the rostral dentitions incline rostrally and gradually change to incline caudally in the caudal part of the tooth row (Fig. 4B). Each dentary tooth occludes between two maxillary crowns. In dorsal view, the dentary tooth rows diverge at a similar angle to the maxillary tooth rows (Fig. 4A).

The morphology of the dentary tooth roots is similar to that of the maxillary teeth, including nearly conical roots with oval cross-sections, inverted V-shaped pits, and open pulp cavities (Fig. 4D). In labial view, the functional crowns develop more prominent primary ridges (Fig. 4B-C). Mesial and distal to the primary ridges all carinae develop four denticles (Fig. 4D). Differing from the maxillary dentitions, the wear facets are positioned on the labial surfaces of the functional crowns distally (Fig. 4B).

Within the same individual, the counts of replacement teeth per side of the dentary are similar to those of the maxillary dentitions although the insertion positions are different (Fig. S3). Only the subadult specimen IVPP V15717 preserves a second generation of replacement teeth in the 12th alveolus of left dentary tooth row (Fig. 4F). Digital reconstructions show that the replacement roots are similar to the functional roots but have thinner dentine (Fig. 4E). The replacement crowns are triangular in lingual view, compressed labiolingually, and the denticles extend along nearly the entire crown margin (Fig. 4D). In the caudal part of the dentary tooth rows, the replacement teeth are positioned more distally which results in more resorption in the distal part of the functional roots (Fig. 4D).

The remnants of resorbed functional teeth are irregular and composed of thin layers of dentine (Fig. 4B-C). These remnants are usually positioned labial to the functional teeth although there is some deviation. The remnants in the caudal part are positioned more distally (Fig. 4E-F).

### Replacement progress in ***Jeholosaurus***

**Fig. 5:**
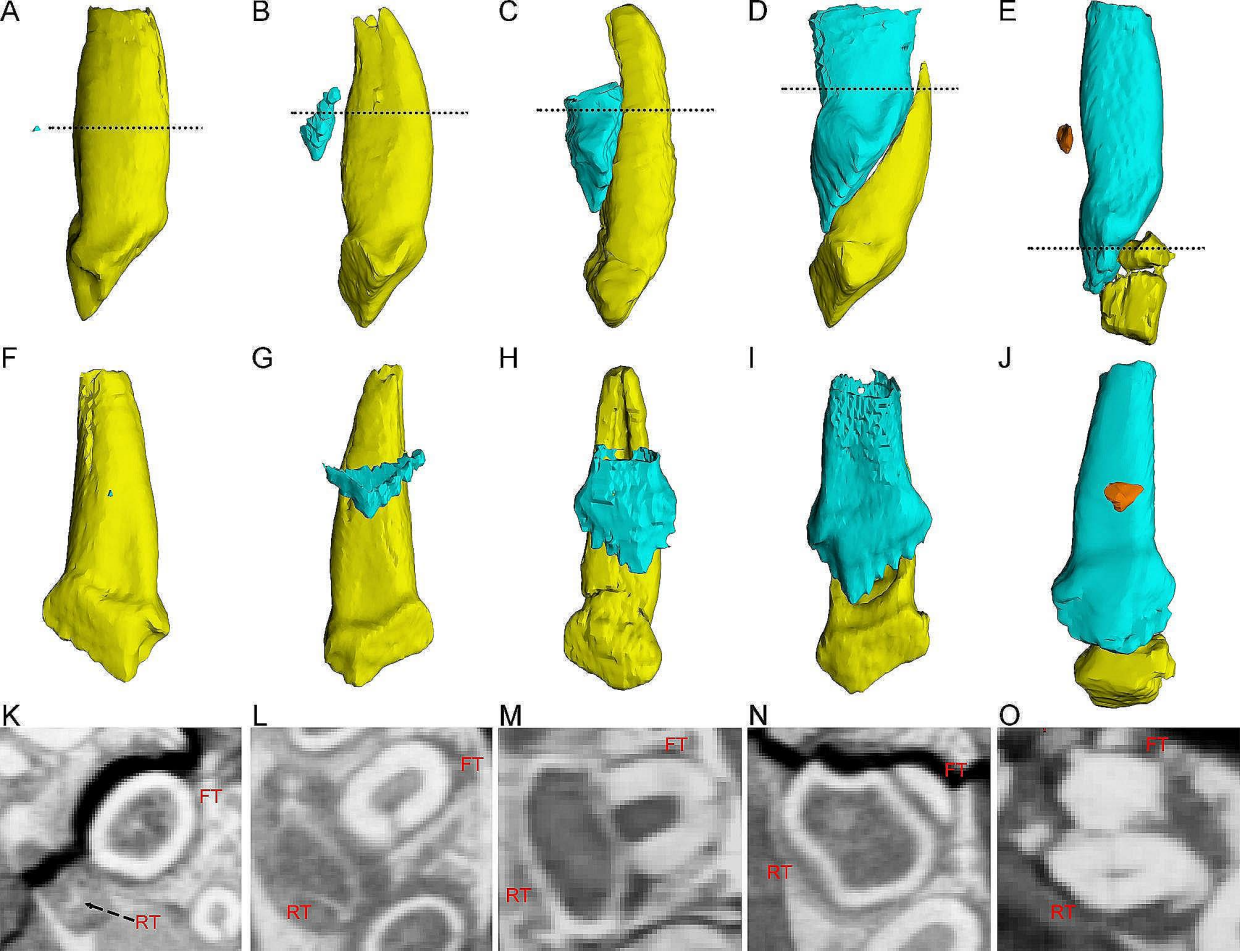
The replacement progress illustrated by teeth at different replacement stages in the maxilla of *Jeholosaurus*. Elements in the CT reconstructions are color coded as follows: functional maxillary teeth, yellow; replacement teeth, cyan. (**A**, **F**, **K**) rM13 in the early juvenile IVPP V15719; (**B**, **G**, **L**) lM10 in the early juvenile IVPP V15719; (**C**, **H**, **M**) rM7 in the late juvenile IVPP V12529; (**D**, **I**, **N**) lM9 in the late juvenile IVPP V15719; (**E**, **J**, **O**) lM10 in the subadult IVPP V15717. **A**-**E**, teeth in mesial and distal view; **F**-**J**, teeth in lingual view; **K**-**O**, teeth in cross-sections. The imaginary lines indicate the position of the cross-sections

In *Jeholosaurus*, tooth replacement begins with germination of the tip of the replacement tooth (Fig. 5A, F). The tip is composed of a thin layer of dentine and positioned level with the middle part of the functional tooth root some distance away from it lingually (Fig. 5A, F). In cross-section, the replacement tooth can be seen to cause resorption of the jaw bone, forming a small slot (Fig. 5K). After a period of growth, the replacement tooth consists of a partial crown represented by small denticles (Fig. 5B, G). The crown remains a small distance from the functional tooth. In this stage, the dentine develops more labially than lingually (Fig. 5L). Subsequently, the replacement crown continues to grow and gradually moves labially and crownward. When grown completely, the replacement crown clings to the functional tooth (Fig. 5C, H). Between these two stages, the lingual dentine of the functional tooth is gradually resorbed and the pulp cavity is opened (Fig. 5M). During ontogeny, the replacement tooth grows crownward, resulting in the tip bracing against the cingulum, and the root forms (Fig. 5D, I). At this moment, the functional root is resorbed to leave a labial thin layer of dentine (Fig. 5N). In most cases, the replacement tooth enters the alveolus and the functional crown would be shed to finish replacement. However, in the subadult specimen (IVPP V15717) the second generation of replacement tooth appears before the functional crown is shed. The functional tooth is resorbed almost entirely leaving only a part of the crown (Fig. 5E, O). The second generation replacement tooth is positioned at the lower part of the root differing from the position of the first generation (Fig. 5J).

### Zahnreihen in ***Jeholosaurus***

**Fig. 6:**
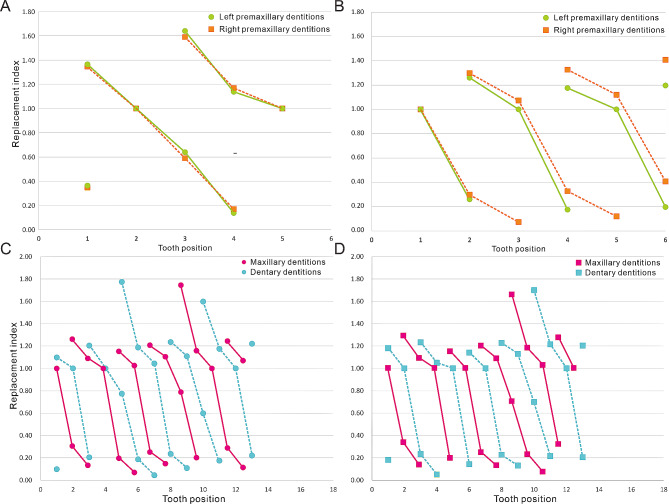
Zahnreihen graphs in *Jeholosaurus* (early juvenile CUGW VH132, IVPP V15719 and subadult IVPP V15717). X-axis is the tooth position, Y-axis is the tooth replacement index. (**A**) premaxillary dentitions of CUGW VH132; (**B**) premaxillary dentitions of IVPP V15717; (**C** and **D**) left and right maxillary dentitions of IVPP V15719. Imaginary and full lines represent the Zahnreihen

The measurements of the reconstructed dentition have been used to calculate the replacement index (Fig. 6 and S4-5). Due to the broken premaxillae and lack of premaxillary teeth, only the early juvenile CUGW VH132 and the subadult IVPP V15717 have reconstructed premaxillary Zahnreihen (Fig. 6A and B). In the premaxillary Zahnreihen plots, these teeth show that the growth stage decreased progressively over several tooth positions. In the early juvenile CUGW VH132, two Zahnreihen are reconstructed on both sides with a Z-spacing of 2.66. The Zahnreihen of both sides are consistent and new teeth erupt from caudal to rostral. However, Zahnreihen in the subadult IVPP V15717 are asynchronous on the left and right sides. The right Zahnreihen are composed of more teeth with a Z-spacing of 2.13. Z-spacing in the left dentition is 1.91 indicating that new teeth erupt in the opposite order in the left [23].

Maxillary dentitions have a different pattern of Zahnreihen from the premaxillary dentitions (Fig. 6, S4). Six to eight Zahnreihen are identified on the right and left maxillae. The longest Zahnreihe is composed of six teeth. In the same individual, Zahnreihen in both maxillae are symmetrical, differing from the premaxillary dentitions. In five smaller specimens, the spacing between Zahnreihen ranges from 2.43 to 2.58 (Table 2). However, the largest specimen has the greatest Z-spacing with 2.83 and 2.71 on the left and right sides, respectively. Overall, Z-spacing in the maxillae ranges from 2.43 to 2.83 and the average is 2.53. The dentary dentitions have a similar tooth replacement pattern to the maxillary dentitions except for the largest specimen (Fig. S5). Five to eight Zahnreihen are identified on each side of the dentary and each Zahnreihe is composed of six teeth at most. In the dentary, Z-spacing is between 2.34 and 2.62 with an average of 2.478 (Table 2).

Demar [46] reported that the Z-spacing ranges from 1.56 to 2.80 in most reptiles, with those of *Jeholosaurus* falling in this range. In some early-diverging ceratopsians, the Z-spacings are greater than 2.0, similar to *Jeholosaurus* [10, 45]. So far, all known Z-spacings in almost all non-avian dinosaurs are also greater than 2.0 [23, 24, 47–49]. However, Z-spacing in the left dentary of *Tenontosaurus* is 1.40 and 1.44 in the left maxilla [50]. *Tenontosaurus*, an early-diverging iguanodontian, is the only known dinosaur with a Z-spacing less than 2.0. Z-spacing greater than 2.0 indicates a replacement wave direction that is rostral to caudal, which is reversed when Z-spacing is less than 2.0, and teeth are replaced in simple alternation between odd- and even-numbered tooth positions when the value is exactly 2.0 [23]. This indicates that new teeth in the maxillae and dentaries erupt from caudal to rostral order in either odd- or even-numbered alveoli. Consequently, new teeth in *Tenontosaurus* erupt in the opposite order to that in other early-diverging ornithopods.

**Table 2:** Z-spacing in *Jeholosaurus*

Specimen number	Premaxillary dentitions		Maxillary dentitions		Dentary dentitions	
	Left	Right	Left	Right	Left	Right
CUGW VH132	2.62	2.69	2.47	2.49	2.58	2.48
IVPP V15719	-	-	2.44	2.43	2.53	2.58
IVPP V12530	-	-	2.52	2.51	2.42	2.45
IVPP V15718	-	-	2.58	2.53	2.60	2.62
IVPP V12529	-	-	2.45	2.44	2.37	2.38
IVPP V15717	1.91	2.13	2.82	2.71	2.38	2.34

Edmund [11] considered that the replacement rhythm is disrupted at the premaxillary-maxillary suture which was also reported in *Tarbosaurus* [23] and *Alligator mississippiensis* [51]. Between the rostral maxillary region and the caudal premaxillary region, a depression is present that results in the interruption of the dental lamina so that the development of the premaxillary and maxillary dentitions are at least partly independent events [51]. The phenomenon also appears in early juvenile (CUGW VH132) and subadult (IVPP V15717) *Jeholosaurus*. In this case, the dentitions in the upper and lower jaws also develop from the different dental lamina. However, we plotted the maxillary and dentary Zahnreihen together and found that the maxillary Zahnreihen could coincide with the dentary Zahnreihen after a certain adjustment (Fig. 6C and D). This suggests that the maxillary dentitions have similar tooth replacement patterns to the dentary dentitions even though they develop from different dental laminae.

### Tooth formation time and tooth replacement rate

**Table 3:** Details of the specimens used for histological study and tooth formation times counted on *Jeholosaurus*

Specimen number	Tooth position	Tooth formation time (days)	DDAR (µm)	Direction of section
YLSNHM01797	rM8	46	11.7178	Coronal
YLSNHM01797	lPM4	33	13.7688	Coronal
CUGW VH132	rPM5	25	12.7401	Mesiodistal

In the coronal thin-sections of rM8 and lPM4 in the subadult YLSNHM01797, we counted incremental lines of von Ebner and found that the tooth formation times are 46 days in the maxilla and 33 days in the premaxilla (Table 3). In the mesiodistal thin-section of rPM5 in the early juvenile CUGW VH132, the tooth formation time is 25 days in the premaxilla (Table 3). This duration is lower than *Hungarosaurus* (63–126 days), *Mochlodon* (77–140 days), *Pinacosaurus* (75 days), *Edmontonia* (279 days), and *Changchunsaurus* (58 days) [52, 53]. In *Triceratops* and hadrosaurs, the mean tooth formation times range from 132 to 381 days [36]. The mean DDARs in the thin-sections of rM8 and lPM4 in the subadult YLSNHM01797 are 11.7178 μm and 13.7688 μm lower than *Changchunsaurus* (19.5 μm) with similar body size [52]. The juvenile CUGW VH132 bears similar DDAR (ranges from 11.7178 to 13.7688) with large specimens about 12.7401 in the thin-section of rPM5 (Table 3).

The tooth replacement rate is calculated as the difference between the number of days recorded in replacement teeth within the same tooth family [54]. However, this method requires destructive sampling of the tooth-bearing bones so we were unable to estimate differences in formation times. In thin-sections, the pulp cavities of the maxillary teeth are open and no obvious tooth resorption is visible (Fig. S6). Therefore, the maxillary tooth is newly erupted and no replacement tooth (or only the replacement tooth bud) was present in this tooth family. This suggests that the tooth replacement rate for *Jeholosaurus* may have been the same as, or slightly less than, the tooth formation time (46 days). The tooth replacement rate in the early juvenile (CUGW VH132) is about or slightly less than 25 days because rPM5 is newly erupted. Hence, it appears that the tooth replacement rate slowed during ontogeny in *Jeholosaurus*, as also reported in *Alligator* [55]. The difference in tooth replacement rate is caused mainly by size differences in view of the similar DDAR. For comparison, the tooth replacement rate is faster than *Mochlodon* (140 days), *Triceratops* (83 days), hadrosaurs (range from 46 to 83 days), and ankylosaurs (53–120 days) [36, 53]. The tooth replacement rate in *Jeholosaurus* is similar to those sauropods with dental batteries [54].

Previously, data on tooth replacement rates in non-iguanodontian ornithopods were limited. Some studies hypothesized that the process and rate of tooth replacement in early-diverging ornithopods were similar to those of thyreophorans [11, 53]. Here, we suggest that the tooth replacement rate in *Jeholosaurus* is faster than that of thyreophorans and, at most, two generations of replacement teeth existed. Tooth formation time in *Changchunsaurus* is relatively short [52] and probably accompanied by a relatively fast replacement rate. However, rhabdodontids (*Mochlodon* and *Matheronodon*) have a slow replacement rate and only one generation of replacement teeth. Late-diverging iguanodontians and hadrosaurs gradually evolved a high replacement rate and more than two generations of replacement teeth. Hence, fast replacement rate and multi-generation replacement teeth have evolved at least two times independently in Ornithopoda (Fig. 8).

## Discussion

### Ontogenetic changes in the dentition of ***Jeholosaurus***

In *Jeholosaurus*, the number of premaxillary teeth increases through ontogeny as also reported in *Thescelosaurus* [56]. In *Thescelosaurus*, six teeth are present in the premaxilla but specimens in early ontogenetic stages have lower tooth counts [56]. Our reconstructions suggest that the maxillary and dentary dentitions in *Jeholosaurus* also increase the number of alveoli as also reported in some other ornithischians [10, 43, 57–59].

**Fig. 7:**
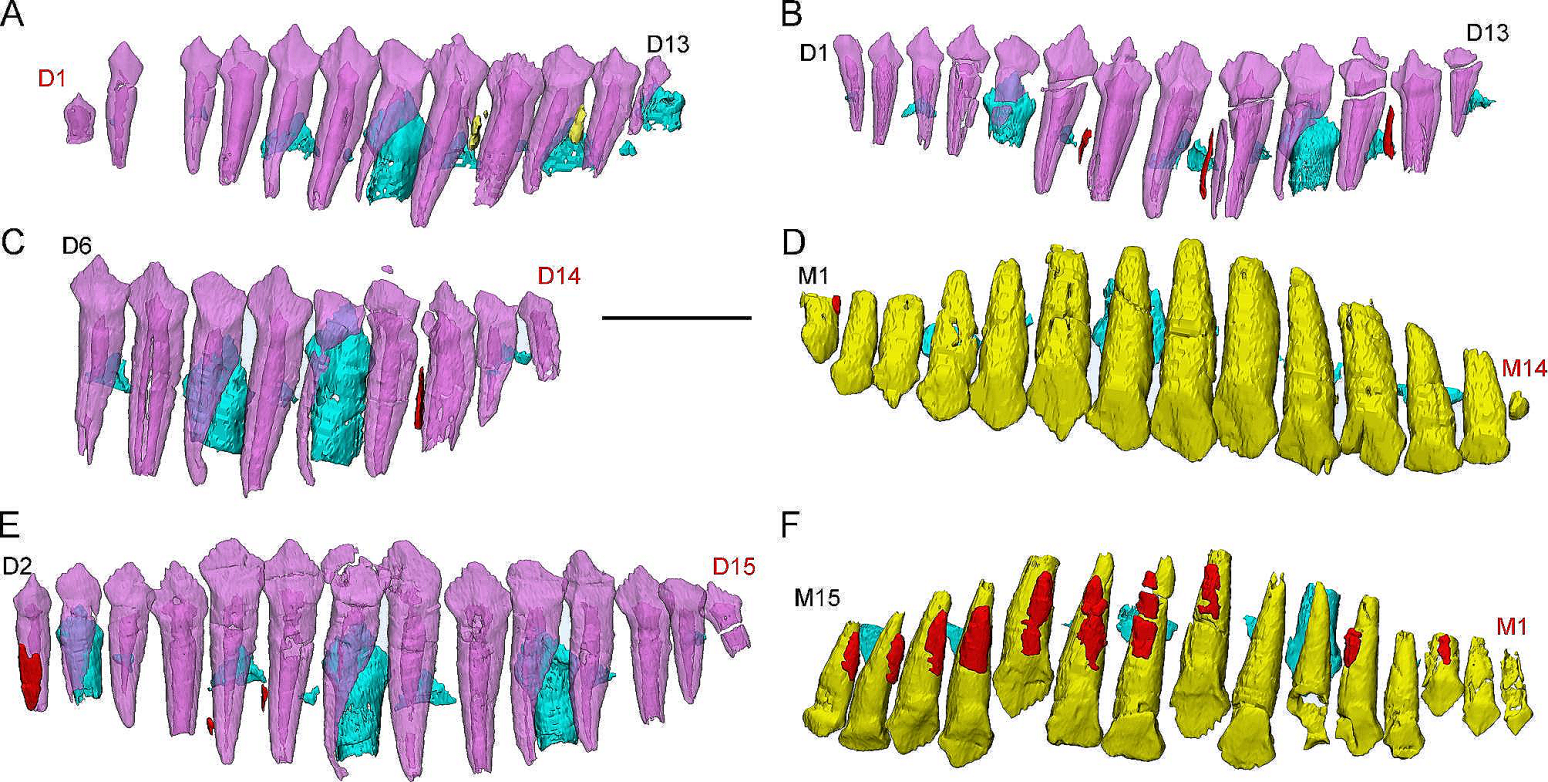
Ontogenetic changes in maxillary and dentary dentitions of *Jeholosaurus*. (**A**) Left dentary tooth row of the early juvenile CUGW VH132 in labial transparent view; (**B**) Left dentary tooth row of IVPP V15719 in labial transparent view; (**C**) Left dentary tooth row of the late juvenile IVPP V12530 in labial transparent view; (**D**) Left maxillary tooth row of the late juvenile IVPP V12530 in labial transparent view; (**E**) Left dentary tooth row of the late juvenile IVPP V15718 in labial transparent view; (**F**) Right maxillary tooth row of the late juvenile IVPP V15718 in labial transparent view. Elements in the CT reconstructions are color coded as follows: functional maxillary teeth, yellow; functional dentary tooth, purple; replacement teeth, cyan; remnants of resorbed functional teeth, red. Red front labels the newly erupted tooth. Scale bar equals 5 mm

Our 3D models of this ontogenetic series help us understand the sequence of eruption. The early juvenile (CUGW VH132) bears 13 teeth in the left dentary and lD1 is short with a large pulp cavity similar to a newly erupted tooth (Fig. 7A). In the early juvenile (IVPP V15719) slightly larger than CUGW VH132, left dentary also bear 13 teeth and lD1 has developed fully (Fig. 7B). Therefore, the 13th tooth germinates rostral to the dentary tooth rows. In the late juvenile IVPP V12530, a little cusp (lM14) is positioned caudal to the left maxillary tooth row (Fig. 7D). We suggested it is a newly formed crown with a root undeveloped. In the dentary, lD14 is also short with a large pulp cavity similar to a newly erupted tooth (Fig. 7C). Hence, the 14th teeth in the dentary and maxillae germinate caudal to the tooth rows. In the late juvenile IVPP V15718, the right maxillary tooth row bears 15 functional teeth and the left maxillary tooth row bears 14 functional teeth (Fig. S2). Based on the symmetry of the tooth replacement pattern, we matched the left and right maxillary teeth and suggested that the rM1 newly erupts and the morphology also supports the deduction (Fig. 7F). In the dentary, lD15 exhibits the features of the newly erupted tooth (Fig. 7E). Therefore, the 15th tooth germinates rostral to the tooth rows in the maxilla and caudal to the tooth rows in the dentary. In general, new alveoli in *Jeholosaurus* germinate rostral and caudal to the tooth rows but with uncertain order.

The Z-spacings of the premaxillary dentitions in the subadult (IVPP V15717) suggest that new teeth on the left and right sides erupt in the opposite order. Although Zahnreihen in the late juvenile (IVPP V12529) can not be reconstructed, the measurements of the replacement teeth (see Additional File 2) also show that the premaxillary dentitions of IVPP V12529 were replaced asynchronously on the left and right sides, similar to IVPP V15717. Therefore, the premaxillary dentitions may change replacement rhythm with ontogeny.

Differing from premaxillary dentitions, the maxillary and dentary dentitions of all specimens maintained a synchronous replacement rhythm on the left and right sides (Figs. S4-5). The numbers of replacement teeth in the upper and lower jaws in the smaller four specimens are similar (Figs. S1-S3; Table. S1). In the late juvenile IVPP V12529, there are 13 or 14 replacement teeth out of 15 functional teeth in the upper and lower jaws. Our reconstructions reveal that the subadult specimen bears a higher ratio of the replacement teeth to functional teeth and only the largest specimen develops a second generation of replacement teeth (Table. S1), as in *Liaoceratops* [45] and *Manidens* [47], but differing from *Yinlong* in which the replacement ratio decreases during ontogeny [10]. The two largest specimens (IVPP V12529 and IVPP V15717) bear many remnants of old functional teeth and a second generation of resorbed teeth.

### The evolution of replacement patterns in Ornithischia

**Fig. 8:**
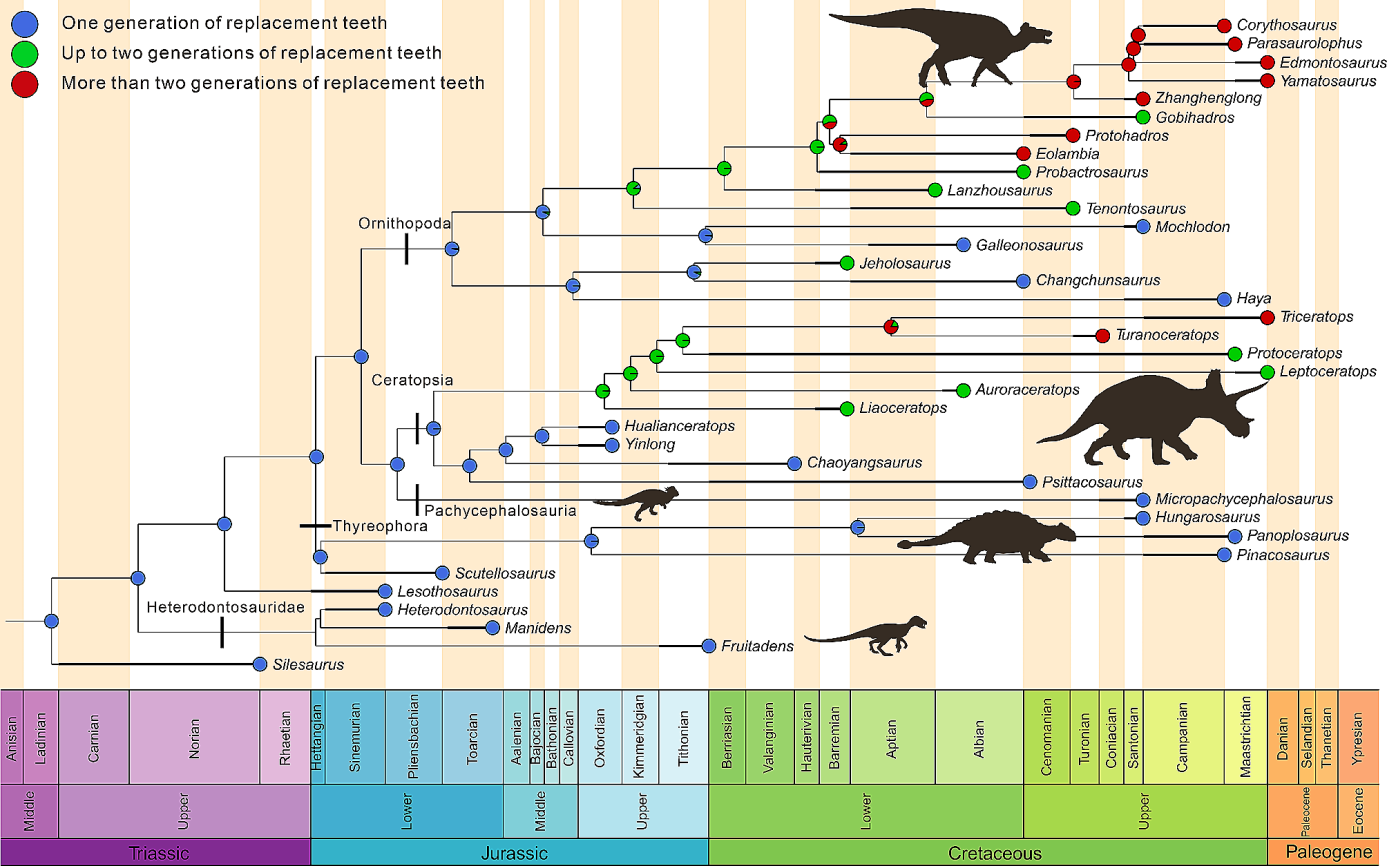
Evolution of the tooth replacement patterns across the ornithischians on calibrated phylogeny using SIMMAP

High-fiber herbivory originated multiple times within ornithischian dinosaurs [1, 4]. However, previous studies have not compared tooth replacement and these independent acquisitions of herbivory. Here, we collated the replacement patterns of the major ornithischian clades and map changes in these character states across the tree. One generation of replacement teeth is reconstructed as the ancestral state for ornithischians by Bayesian stochastic character mapping (Fig. 8) and comparison with the outgroup [23, 60].

In *Lesothosaurus*, one specimen possesses a broken left mandible that bears only two replacement teeth out of eight scanned teeth while a second specimen bears the right maxilla containing 13 functional teeth with one replacement tooth [61, 62]. Sciscio, Knoll [62] inferred that tooth replacement was an asynchronous and slow process in mature *Lesothosaurus*, which is known as the earliest ornithischian with asynchronous tooth replacement. Meanwhile, rare replacement teeth in *Lesothosaurus* are coincident with its facultative omnivorous lifestyle and relatively low bite force [4, 62].

In the earliest diverging ornithischian clade [17, 18, 63], Heterodontosauridae, the replacement patterns have been studied in detail by CT analysis [47, 64, 65]. The holotype of *Fruitadens* includes a left maxilla containing six tooth positions with two replacement teeth and a right dentary containing eight tooth positions with three replacement teeth [64]. Other broken fragments also show evidence of tooth replacement but bear fewer replacement teeth. In the later-diverging heterodontosaurid *Manidens*, the right maxilla of the holotype bears eight functional teeth with five replacement teeth and the left maxilla of another specimen bears 10 tooth positions with two replacement teeth [47]. In the dentaries of *Manidens*, there are 11 tooth positions with seven replacement teeth on the left and six replacement teeth on the right [47]. The difference in the number of functional teeth and the variable ordering of different stages of replacement teeth between dentaries indicate an asynchronous tooth replacement between the left and right dentary dentitions of *Manidens*. In *Heterodontosaurus tucki*, the posterior region of the left maxilla preserves seven functional teeth with three replacement teeth [65]. However, evidence of continuous tooth replacement appears to be absent in both adult and juvenile specimens [66]. Similar to *Lesothosaurus*, heterodontosaurids bear only one generation of replacement teeth but with relatively more replacement teeth per side of the jaw. Button, Porro [4] suggested that *Heterodontosaurus*, which has low mechanical advantage values at every biting position, achieved elevated bite forces through larger adductor muscles relative to skull size. Therefore, a fast replacement rate at each alveolus is not necessary for heterodontosaurids that relied instead on more specialized dentitions (e.g., closely packed high-crowned teeth, ventral deflection of the jaw joint, enhanced adductor musculature) for processing tough plant material.

In another major ornithischian clade, thyreophorans, the replacement patterns are poorly understood. Using nanoCT scanning, a right dentary of *Hungarosaurus* was shown to contain 16 alveoli with about 10 replacement teeth and only one generation of replacement teeth [53]. Some other thyreophorans (*Scutellosaurus*, *Pinacosaurus*, and *Panoplosaurus*) with detached tooth-bearing bones also have one generation of replacement teeth at most [3, 67, 68]. The replacement patterns of thyreophorans seem to be poorly adapted for habitually processing high-fiber plant matter, especially in ankylosaurs where complex jaw mechanisms are demonstrated by tooth morphology and wear [3]. The early-diverging thyreophoran *Scelidosaurus* exhibits increased absolute biting performance due to relatively greater body size [4]. In late-diverging thyreophoran lineages, precise tooth occlusion [69] and extensive gut fermentation [2], alongside large body size, help in processing food.

At present, the tooth replacement pattern in early-diverging ornithopods has not been described in detail. Transverse thin-sections through the dentaries of *Changchunsaurus parvus* have revealed that about nine replacement teeth exist for the 15 tooth positions [52]. According to our reconstructions, the counts of replacement teeth in *Jeholosaurus* per side of the jaw are slightly more than that in *Changchunsaurus*. However, dental histology may obscure some replacement tooth buds. These two taxa may bear similar tooth replacement patterns which correspond to their close phylogenetic positions. Researchers conducted CT analysis on another early-diverging ornithopod, *Haya griva*, and found that the maxilla (IGM 100/2017) contains 14 alveoli whereas the left contains 13 [70]. The maxillae contain five replacement teeth on the left side and eight on the right, which is less than either *Jeholosaurus* or *Changchunsaurus*. In the right dentary, CT scans reveal that the five erupted teeth have single replacement teeth among 16 alveoli [70]. The degree of formation of the replacement teeth differs between the left and right sides which suggests that the timing of the replacement waves differed as is the case for the dentaries in *Haya* [70]. External observations (i.e. in the absence of CT data) of the later-diverging ornithopods *Parksosaurus* [71] and *Hypsilophodon* [72] suggest that tooth replacement alternates by approximately every other tooth and the number of replacement teeth may be higher. In general, early-diverging ornithopods bear similar numbers of replacement teeth and only *Jeholosaurus* preserves a second generation of replacement teeth. In the iguanodontian *Tenontosaurus* a total of 13 vertical tooth families is present with a maximum of two teeth present in each. There are 14 tooth sockets in the dentaries with three teeth in four families and two in the rest [50]. *Tenontosaurus* has a higher ratio of replacement teeth to functional teeth and more replacement teeth per side of the jaw under the second generation than *Jeholosaurus*. Previously, *Tenontosaurus* was the earliest-diverging ornithopod with a second tooth in each alveolus. The presence of a second generation of the replacement teeth in *Jeholosaurus* widens the phylogenetic distribution of this characteristic. In other early-diverging ornithopods, such as *Changchunsaurus*, no second replacement teeth have been found, but it is possible that these taxa have not yet been studied in sufficient detail or that the available specimens are from ontogenetic stages that are too early to preserve them. Ornithopods more derived than *Iguanodon bernissartensis* (including all hadrosaurs) are usually thought to bear two or more teeth in each alveolus [50]. In general, the evolution of dentitions from the earliest-diverging ornithopods to hadrosaurs includes the increase of teeth in each tooth family and an increase in the number of replacement teeth present at any one time.

The replacement pattern in ceratopsians has been researched in detail [10, 45]. In early-diverging groups, each alveolus bears at most one replacement tooth indicating lower replacement rates than late-diverging ceratopsians. In early-diverging neoceratopsians (*Liaoceratops* and *Auroraceratops*), an alveolus bears at most two replacement teeth with a relatively lower replacement rate [45, 73]. *Protoceratops* and ceratopsids bear two or more replacement teeth in each alveolus.

In summary, the replacement patterns evolved in parallel between Ornithopoda and Ceratopsia with increasing numbers and generations of replacement teeth and the formation of dental batteries. Although significant functional differences occurred between the early-diverging members of these clades, like *Psittacosaurus* and *Hypsilophodon*, greater mechanical efficiency and bite forces are predicted in these two clades than in other clades [4]. In Ornithopoda and Ceratopsia, the number of replacement teeth increased from the Early Cretaceous. The convergent changes of tooth replacement patterns between these two clades are accompanied by the increased mechanical advantage in the jaw system [74–76]. Relative weak jawed thyreophorans show only one generation of replacement teeth. The jaw apparatus and the replacement patterns are highly coupled in ornithischians. The evolution of sophisticated oral processing in these clades is not only associated with a suite of craniodental and myological characters but also changes in tooth replacement patterns and these clades evolved different replacement patterns for processing high-fiber plant materials.

## Conclusions

An ontogenetic series of *Jeholosaurus* specimens provides a good opportunity to understand the ontogenetic changes of the dental system in Ornithopoda. Ontogenetic changes in *Jeholosaurus* include increasing numbers of alveoli in the upper and lower jaws, increasing numbers of replacement teeth, the presence of a second generation of replacement teeth, and an increasing number of resorbed functional teeth. Reconstructions of Zahnreihen suggest that the replacement patterns in the maxillary and dentary dentitions are the same but differ from those in the premaxillary tooth row. In addition, the replacement patterns change with the ontogeny, such as the inversion of the replacement wave in the premaxillary dentitions and a transition from synchronous replacement to asynchronous replacement. These changes suggest that the replacement rate in *Jeholosaurus* slowed during ontogeny.

The replacement patterns reveal limited convergence among ornithischians that faced similar ecological pressures. Thyreophorans and heterodontosaurids exhibit relatively fewer replacement teeth and only one generation of replacement teeth. These taxa adapted to high-fiber feeding through increased body size or the evolution of more specialized dentitions. Ornithopods and ceratopsians exhibited similar solutions to the challenges of herbivory through increasing numbers of both replacement teeth and generations of replacement teeth.

### Electronic supplementary material

Below is the link to the electronic supplementary material.

Supplementary Material 1

Supplementary Material 2

Supplementary Material 3

Supplementary Material 4

Supplementary Material 5

Supplementary Material 6

Supplementary Material 7

Supplementary Material 8

Supplementary Material 9

Supplementary Material 10

## Data Availability

All data generated or analyzed during this study are included in this published article [and its supplementary information files].

## Abbreviations

IVPP: Institute of Vertebrate Paleontology and Paleoanthropology, Beijing, China
CUGW: China University of Geosciences (Wuhan), Wuhan, China
YLSNHM: Yingliang Stone Natural History Museum, Fujian, China
lPM and rPM: left and right functional tooth in the premaxilla
lM and rM: left and right functional tooth in the maxilla
lD and rD: left and right functional tooth in the dentary
RPM: premaxillary replacement tooth
RM: maxillary replacement tooth
RD: dentary replacement tooth
SRT: second generation of replacement teeth
iv: inverted V-shaped pit
lc: longitudinal sulci
OF: remnant of the old resorbed functional tooth
pc: pulp cavity
wf: wear facets
sm: serrated margin
