# Supplementary material for: Tooth replacement in the early-diverging neornithischian Jeholosaurus shangyuanensis and implications for dental evolution and herbivorous adaptation in Ornithischia
Source: BMC Ecol Evol. 2024 Apr 16;24:46. doi: 10.1186/s12862-024-02233-2 (PMC11020315; doi:10.1186/s12862-024-02233-2)
Supplement: Supplementary file 3 — Supplementary Material 3 [file 12862_2024_2233_MOESM3_ESM.docx]

**Fig.** **S1** Schematic representation of the dentitions in the premaxillae of four *Jeholosaurus* specimens.

(A). CUGW VH132 (early juvenile); (B). IVPP V12530 (late juvenile); (C). IVPP V12529 (late juvenile); (D). IVPP V15717 (subadult). Open circles, the functional teeth; red circles, the replacement teeth; grey circles, the broken teeth. Each circle is plotted as the ratio of the length of each tooth to that of the longest tooth in the jaw multiplied by 5.0 mm. The replacement teeth have been precisely located concerning the corresponding teeth they will replace.

**Fig. S2** Schematic representation of the dentitions in the maxillae of six *Jeholosaurus shangyuanensis* specimens.

(A) CUGW VH132 (early juvenile); (B) IVPP V15719 (early juvenile); (C) IVPP V12530 (late juvenile); (D) IVPP V15718 (late juvenile); (E) IVPP V12529 (late juvenile); (F) IVPP V15717 (subadult). Open circles, the functional teeth; red circles, the reacement teeth; grey circles, the broken teeth; blue circles: the second generation of replacement teeth. Each circle is plotted as the ratio of the length of each tooth to that of the longest tooth in the jaw multiplied by 5.0 mm. The replacement teeth have been precisely located concerning the corresponding teeth they will replace.

**Fig. S3** Schematic representation of the dentitions in the dentary of six *Jeholosaurus shangyuanensis* specimens.

(A) CUGW VH132 (early juvenile); (B) IVPP V15719 (early juvenile); (C) IVPP V12530 (late juvenile); (D) IVPP V15718 (late juvenile); (E) IVPP V12529 (late juvenile); (F) IVPP V15717 (subadult). Each circle is plotted as the ratio of the length of each tooth to that of the longest tooth in the jaw multiplied by 5.0 mm. The replacement teeth have been precisely located concerning the corresponding teeth they will replace.

**Fig. S4** Z-spacing diagrams in the maxillae of *Jeholosaurus*. X-axis is the tooth position, Y-axis is the tooth replacement index.

(A and B) left and right Zahnreihen in the early juvenile CUGW VH132; (C and D) left and right Zahnreihen in the early juvenile IVPP V15719; (E and F) left and right Zahnreihen in the late juvenile IVPP V12530; (G and H) left and right Zahnreihen in the late juvenile IVPP V15718; (I and J) left and right Zahnreihen in the late juvenile IVPP V12529; (K and L) left and right Zahnreihen in the subadult IVPP V15717. Black line: left Zahnreihen; Red line: right line. Abbreviations: L FT: left functional teeth; L RT: right functional teeth; L RT: left replacement teeth; R RT: right replacement teeth; 2nd RT: second generation of replacement tooth.

**Fig. S5** Z-spacing diagrams in the dentary of *Jeholosaurus*. X-axis is the tooth position, Y-axis is the tooth replacement index.

(A and B) left and right Zahnreihen in the early juvenile CUGW VH132; (C and D) left and right Zahnreihen in the early juvenile IVPP V15719; (E and F) left and right Zahnreihen in the late juvenile IVPP V12530; (G and H) left and right Zahnreihen in the late juvenile IVPP V15718; (I and J) left and right Zahnreihen in the late juvenile IVPP V12529; (K and L) left and right Zahnreihen in the subadult IVPP V15717. Black line: left Zahnreihen; Red line: right line. Abbreviations: L FT: left functional teeth; L RT: right functional teeth; L RT: left replacement teeth; R RT: right replacement teeth; 2nd RT: second generation of replacement tooth.

**Fig. S6** Histological thin-sections of the teeth of *Jeholosaurus* (the subadult YLSNHM01797 and the early juvenile CUGW VH1232) showing DDAR and the numbers of incremental lines of von Ebner.

(A and D) the maxillary tooth (coronal sections). (B and E) the premaxillary tooth (coronal sections). (C and F) the rPM5 in CUGW VH1232 (mesiodistal sections). The red arrows indicate the incremental lines of von Ebner.
