# Supplementary material for: Tooth replacement in the early-diverging neornithischian Jeholosaurus shangyuanensis and implications for dental evolution and herbivorous adaptation in Ornithischia
Source: BMC Ecol Evol. 2024 Apr 16;24:46. doi: 10.1186/s12862-024-02233-2 (PMC11020315; doi:10.1186/s12862-024-02233-2)
Supplement: Supplementary file 4 — Supplementary Material 4 [file 12862_2024_2233_MOESM4_ESM.docx]

**TABLE S1**. List of the ontogenetic difference in specimens of *Jeholosaurus*.

| Number | Alveoli | | | | | | Replacement teeth | | | | | | Resorbed functional teeth | | | | | |
| --- | --- | --- | --- | --- | --- | --- | --- | --- | --- | --- | --- | --- | --- | --- | --- | --- | --- | --- |
|  | PM | | M | | D | | PM | | M | | D | | PM | | M | | D | |
|  | L | R | L | R | L | R | L | R | L | R | L | R | L | R | L | R | L | R |
| CUGW VH132 | 5 | 5 | 13 | 13 | 14 | 13 | 3 | 3 | 9 | 9 | 11 | 9 | 1 | 1 | 1 | 1 | 2 | 1 |
| IVPP V15719 | - | - | 13 | 13 | 13 | 13 | - | - | 10 | 9 | 10 | 9 | - | - | 1 | 0 | 3 | 3 |
| IVPP V12530 | 6 | 6 | 14 | 13 | 14 | 14 | 1 | 2 | 8 | 7 | 5 | 10 | 0 | 1 | 1 | 3 | 1 | 1 |
| IVPP V15718 | - | - | 15 | 15 | 15 | 15 | 0 | 2 | 11 | 8 | 10 | 11 | 0 | 0 | 0 | 0 | 0 | 3 |
| IVPP V12529 | 6 | 6 | 15 | 15 | 15 | 15 | 4 | 4 | 13 | 14 | 13 | 13 | 0 | 1 | 9 | 9/1 | 3 | 4 |
| IVPP V15717 | 6 | 6 | 18 | 18 | 17 | 16 | 3 | 5 | 16/1 | 15 | 15/2 | 16/1 | 1 | 1 | 11 | 9 | 4 | 2 |

- = not preserved

Red font means the second generation of replacement teeth or resorbed functional teeth.
