# Supplementary figures and images for: Tooth replacement in the early-diverging neornithischian Jeholosaurus shangyuanensis and implications for dental evolution and herbivorous adaptation in Ornithischia
Source: BMC Ecol Evol. 2024 Apr 16;24:46. doi: 10.1186/s12862-024-02233-2 (PMC11020315; doi:10.1186/s12862-024-02233-2)

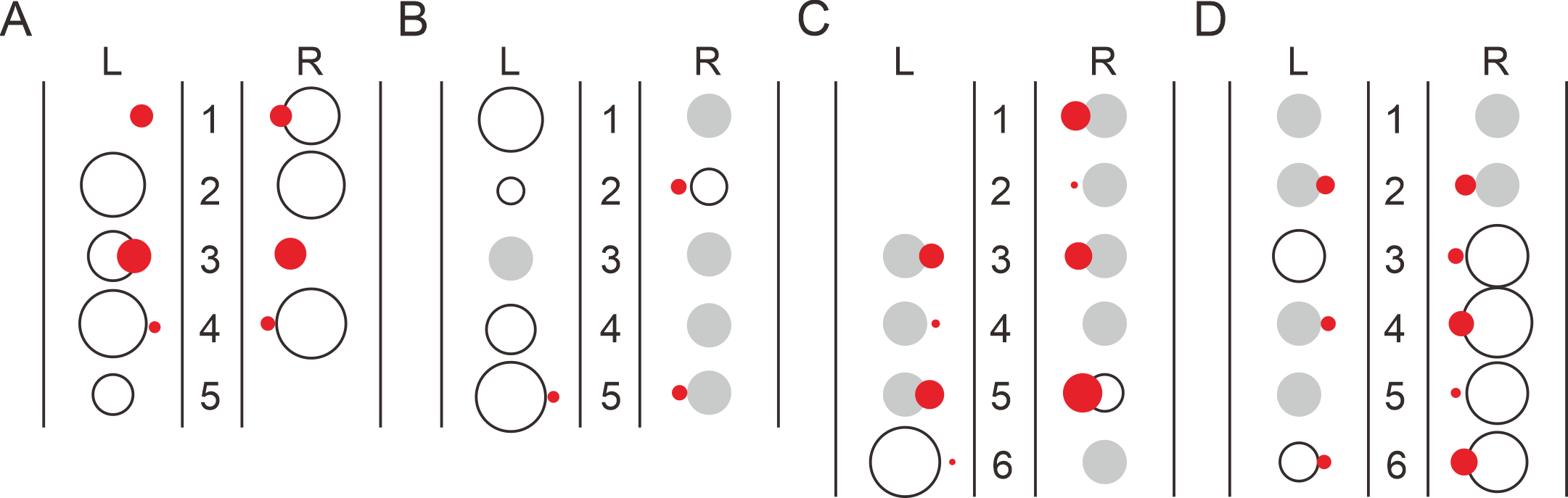

Supplement: Supplementary file 5 — Supplementary Material 5 [file 12862_2024_2233_MOESM5_ESM.png]

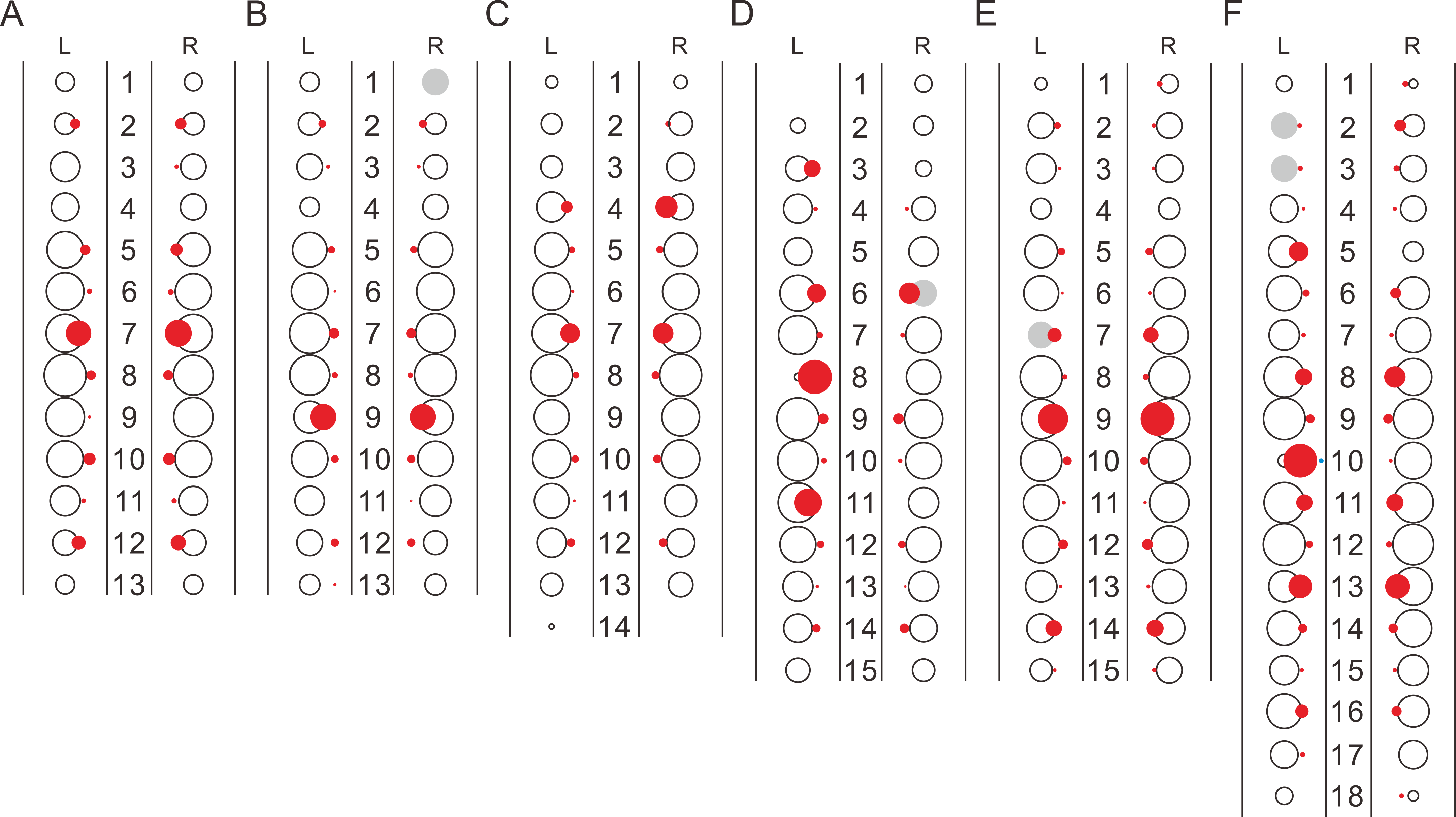

Supplement: Supplementary file 6 — Supplementary Material 6 [file 12862_2024_2233_MOESM6_ESM.png]

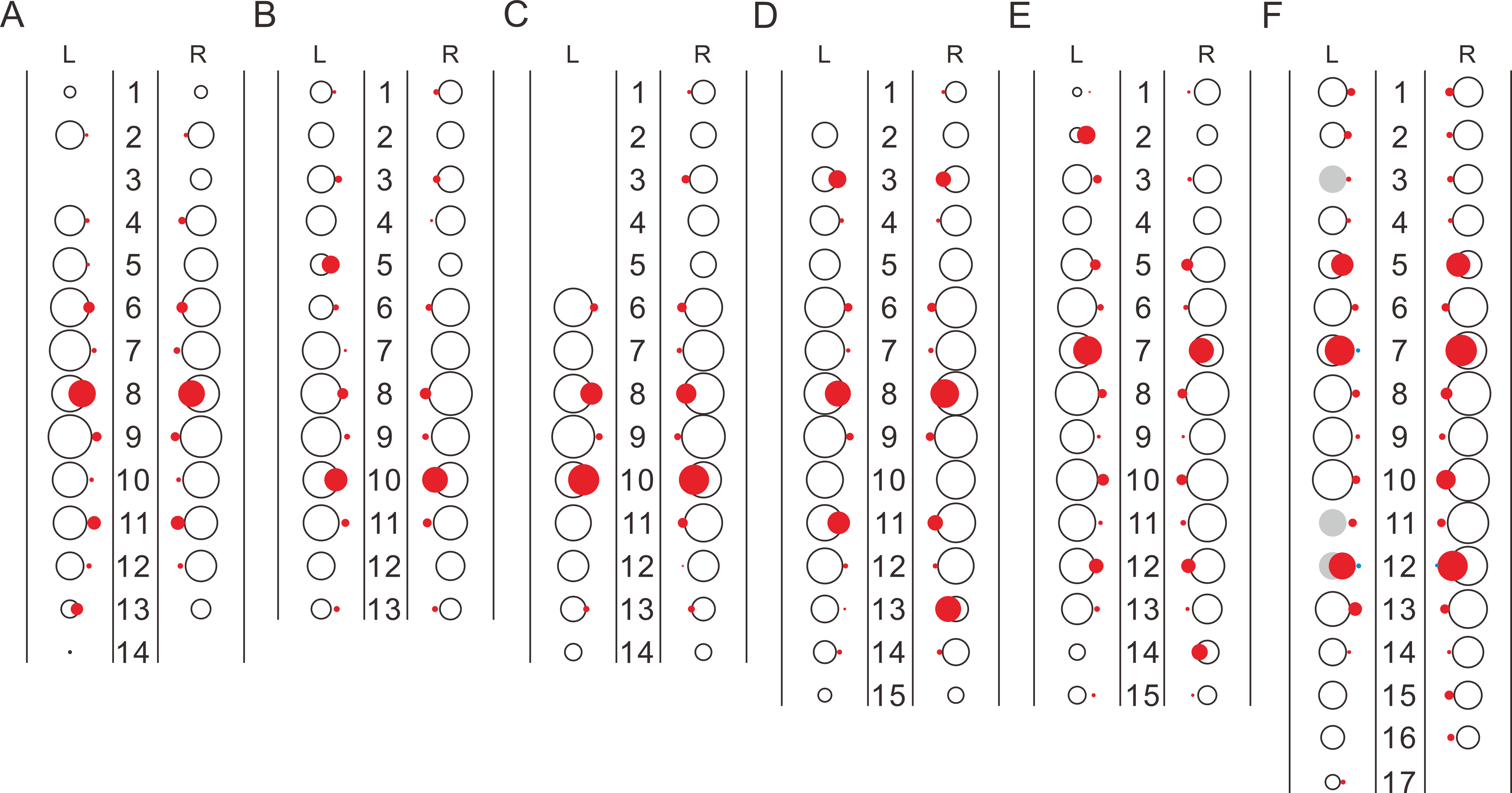

Supplement: Supplementary file 7 — Supplementary Material 7 [file 12862_2024_2233_MOESM7_ESM.png]

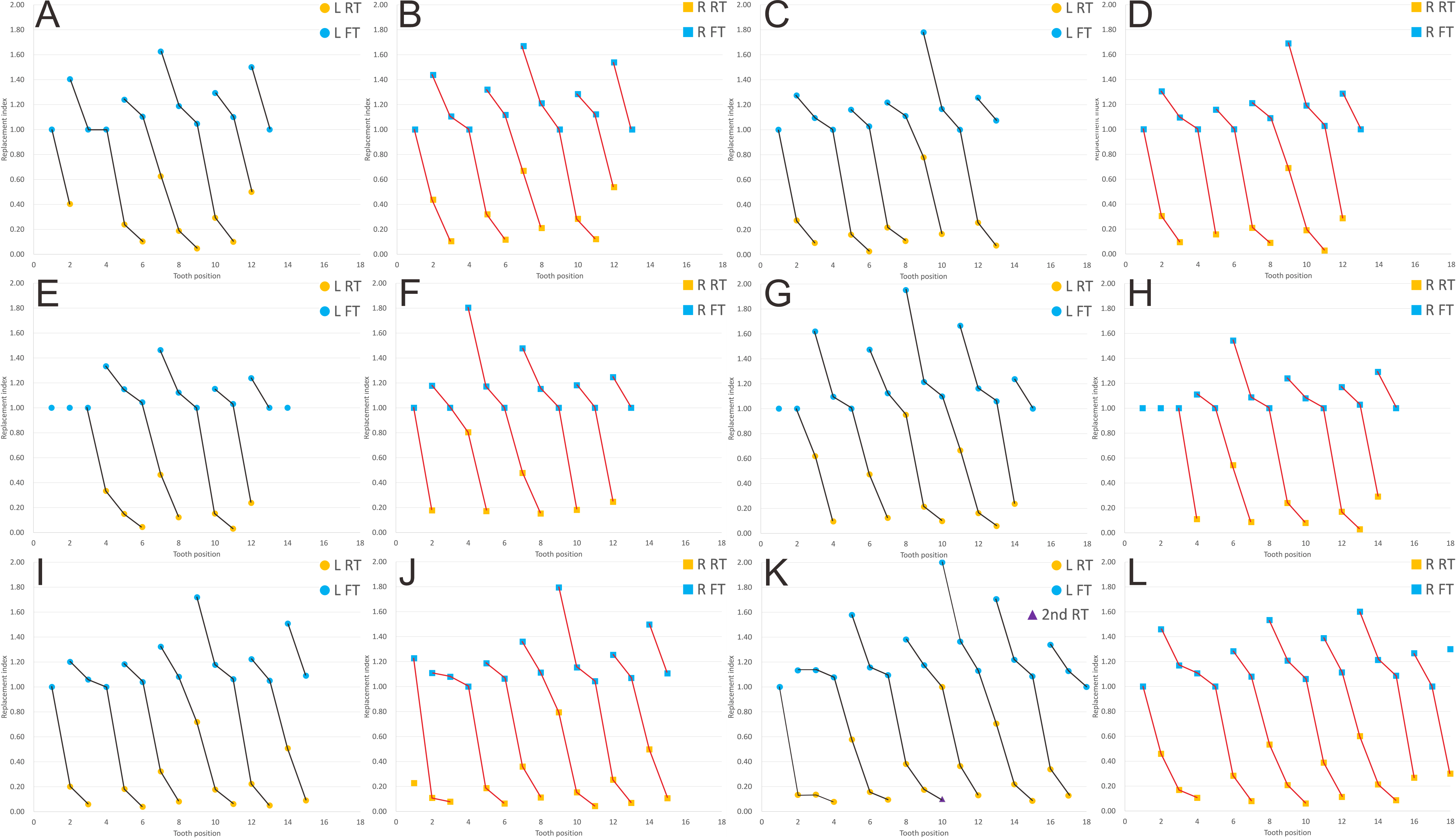

Supplement: Supplementary file 8 — Supplementary Material 8 [file 12862_2024_2233_MOESM8_ESM.png]

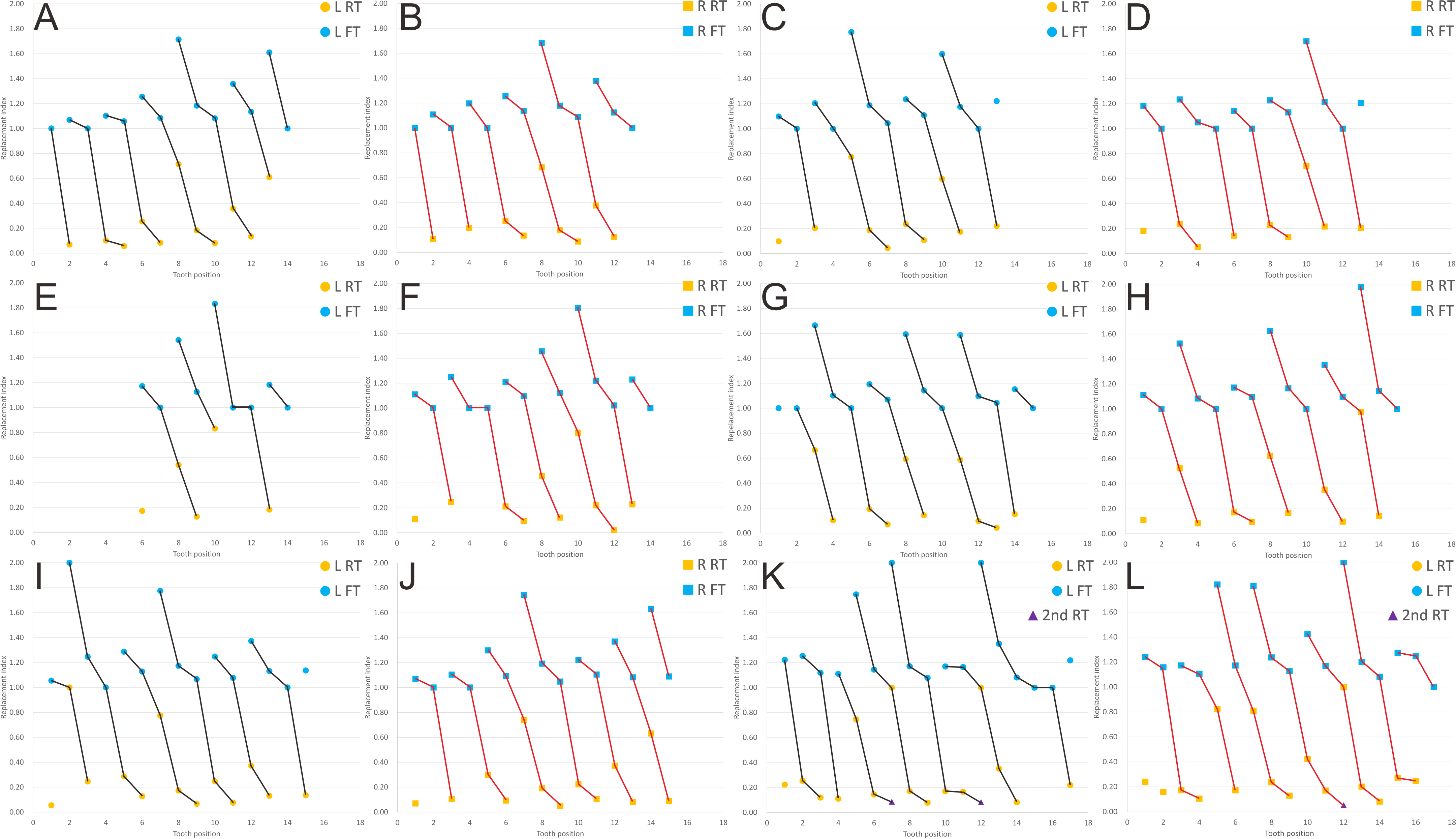

Supplement: Supplementary file 9 — Supplementary Material 9 [file 12862_2024_2233_MOESM9_ESM.png]

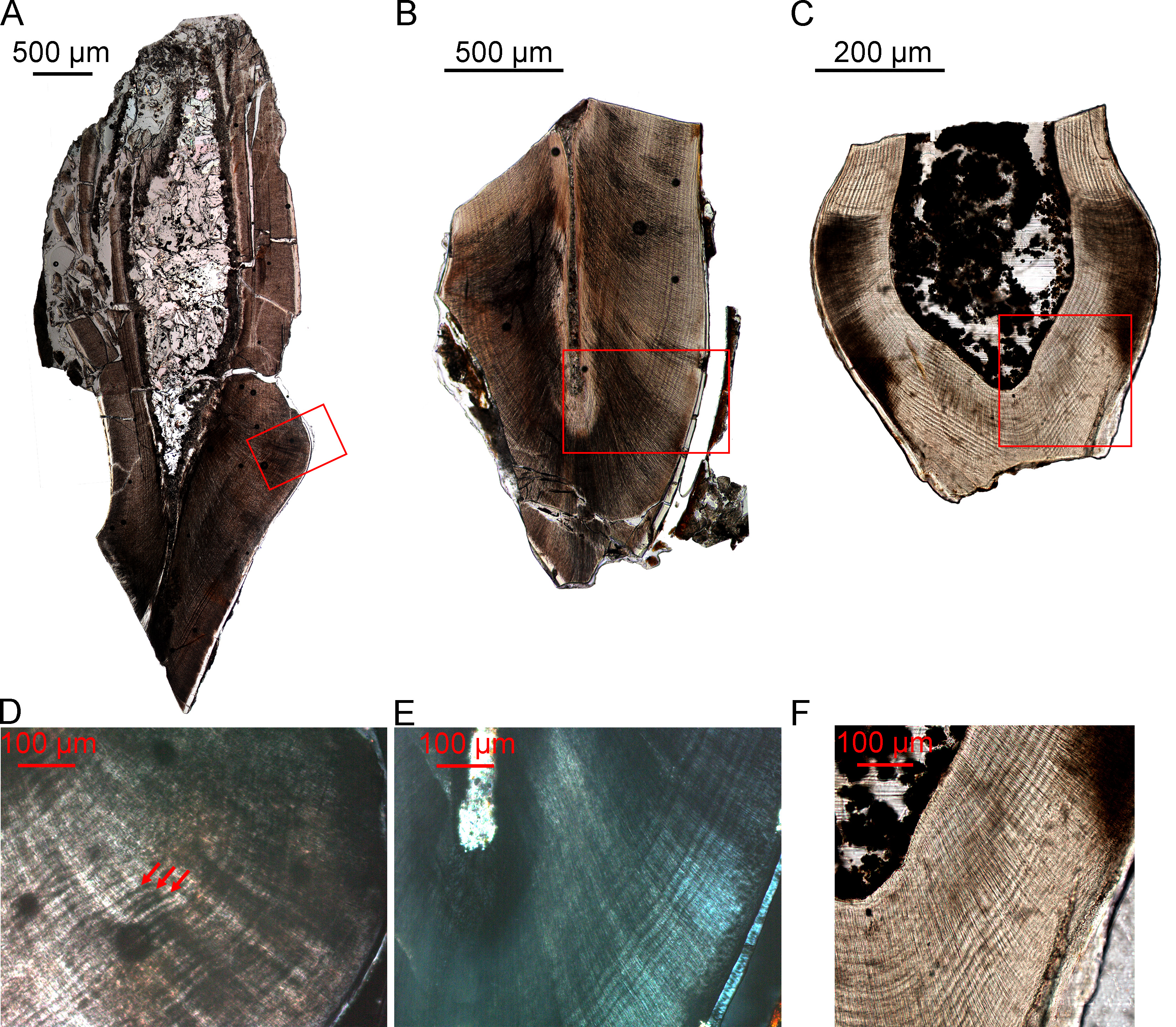

Supplement: Supplementary file 10 — Supplementary Material 10 [file 12862_2024_2233_MOESM10_ESM.jpg]
